# Clean Milk Advice

**Published:** 1920-05-15

**Authors:** 


### CLEAN MILK ADVICE.

THE pure milk film "From Cow to Consumer" (to which we have previously made reference) was shown in Manchester last week, and its presentation was prefaced by a short address from Mr. Buckley, the director of milk supplies to the Ministry of Food. He said the public showed little appreciation of the food value of milk. Even at the higher prices at which it had been sold this winter milk was one of the cheapest foods that could be bought. Under the Government's new system of grading the best milk obtainable was graded "A Certified." This milk would come from a herd certified free from any taint of tuberculosis, and would be bottled on the farm. The next best quality was graded "A." This also would come from a herd free from tuberculosis, but the bottling took place on the retailer's premises. It is understood that during next week or the week after Manchester will receive its first supply of Grade A Certified. One Manchester hospital is already receiving a Grade A supply. "Failing these two ideals of clean milk," Mr. Buckley said, "it seems to me that the only safe thing is to pasteurise. It must be borne in mind, however, that pasteurisation does not atone for filth. Pasteurisation does not clean the milk." He went on to point out that the two grades of milk he had named would necessarily cost more than milk sold under the present system, but they are of enormous value in regard to infant welfare. For many years to come the only thing that can be done with the bulk of milk now sold by open delivery is to pasteurise it, which will kill at least 98 per cent. of the bacteria. Great care must be taken to have the bottles containing the milk properly sterilised and efficiently capped.

